# Wavelengths and Intensities in the First Spectrum of Bromine, 2000 to 13000 A

**DOI:** 10.6028/jres.065A.019

**Published:** 1961-06-01

**Authors:** Jack L. Tech, Charles H. Corliss

## Abstract

The first spectrum of bromine, Br I, has been newly investigated using electrodeless discharge tubes as light sources. The observations have led to a list of wavelengths and estimated intensities of 1056 lines emitted by neutral atoms in the region from 12965 A in the infrared to 3325 A in the ultraviolet. Most of the wavelengths are given to 0.01 A and the intensities are estimated on a relative scale between 1 and 75,000. Lines of Br I were not found in the ultraviolet between 3325 and 1700 A.

## 1. Introduction

Electrodeless discharge tubes containing an iodide or bromide of an element are now widely used as light sources in the study of spectra. In particular, such lamps containing halides are frequently used in this and other laboratories in the study of the spectra of the rare-earth group of elements. An increasing need has therefore been felt for accurate line lists of the spectra emitted by iodine and bromine, in order that wavelengths emitted by these atoms can be successfully deleted from the line list of whatever other element is under investigation. For this reason, and because the halogen spectra present many problems interesting in their own right, this laboratory undertook a program of extending and improving the descriptions and analyses of the arc and first spark spectra of both iodine and bromine. Results of the research on the first spectrum of iodine have already appeared [1] and the results for the first spark spectrum are now in press [2]. The spectrum of singly ionized bromine, Br II, has been observed in the vacuum ultraviolet region as well as in much the visible region, and work on this spectrum is continuing.

The most comprehensive list of Br I wavelengths hitherto available is that contained in the excellent paper on the structure of the arc spectrum of bromine published thirty years ago by C. C. Kiess and T. L. deBruin [3]. Improvements in apparatus and experimental procedures have now permitted a considerable augmentation of this earlier work. Specifically, we have been able to increase the number of lines observed in the spectra region described here to 1056 compared with the 330 lines reported by Kiess and deBruin. It must be mentioned that extensive observations have been completed also in the vacuum ultraviolet and the far infrared regions of the spectrum. These data, together with some Zeeman-effect observations, have already resulted in a well advanced analysis of the first spectrum of bromine and in some revisions of its term structure as reported by Kiess and deBruin. Since this work on the analysis, however, must be temporarily interrupted, it seemed advisable to report separately at this time the complete description of the spectrum in the range from 12965 A to 3325 A, especially in view of its probable usefulness to workers in other laboratories who are using bromides for excitation in electrodeless discharge tubes. The other data, together with the completed analysis of the first spectrum of bromine will be reported at a later time.

## 2. Experimental Procedure

The wavelengths of Br I presented here are derived from two essentially independent sets of observations made at two different times at the Bureau. The first series of observations was made several years ago using as light source an electrodeless discharge tube of the type described by Corliss, Westfall, and Bozman [4]. The vicious attacking of hot metal internal electrodes by the chemically very active bromine, which plagued many early observers of this spectrum, was thus avoided.

The electrodeless tube contained BeBr_2_, produced by evacuating of air a lamp blank containing Be filings and then heating the filings after bromine vapor had been admitted into the blank. To obtain the spectrograms, these lamps were excited in a field of 2,450 Mc/s provided by a microwave oscillator. Spectrograms were made in the first and, where possible, the second orders of 7,500, 15,000, and 30,000 lines per inch concave gratings mounted in parallel light (Wadsworth mounting) at first order reciprocal dispersions of 10, 5, and 2.4 A/mm respectively. Eastman Kodak spectroscopic plates of types 103a–O, 103a–F, I–N, I–Q, and I–Z were used in the appropriate regions.

The line list thus obtained was regarded as fairly complete until the fall of 1959 when W. F. Meggers pointed out that some bromine lines of intensity one in our list appeared much more strongly than that on some plates he was measuring which had been exposed to a lamp containing YbBr. It was then decided to prepare some new lamps and to obtain at least one more set of observations in the entire range of wavelengths. In fact, however, except for the region extending from 4264 to 5721 A and except for the fainter new lines, at least two new measurements were made on each line. The lamps used in this second series of observations were also of the electrodeless type. They contained, however, not a salt, but bromine vapor at a few millimeters pressure. The bromine used was of ACS purity. To avoid the presence of objectionable water vapor in the lamps, which also had often been a source of annoyance in the early observations of the spectrum, the bromine was thoroughly dried. This drying was accomplished on a vacuum system by means of a U-tube terminated at both ends by stopcocks and leading on one side to a reservoir into which bromine had been admitted and on the other side to another reservoir, the lamp blank, and finally the pumps. The U-tube was filled in a dry-box with P_2_O_5_. The bromine vapor made several passes through the drying agent before it was finally collected in the lamp blank. By setting up the microwave oscillator at the vacuum system, the pressure of bromine vapor was adjusted until the most complete arc spectrum was obtained, as judged by observing the discharge visually through a small spectroscope. The pressure was of the order of several millimeters of mercury and the discharge exhibited a reddish color. At much below this optimum pressure the discharge changed to a white color, and there appeared a continuous emission spectrum interrupted only by the strongest Br I lines and apparently arising from the Br_2_ molecule. In the other direction, as the pressure was increased above the optimum pressure, the spectrum remained pure, but the length of the discharge became shorter and eventually the lamp ceased to operate. This same behavior at lower pressures could be observed after the lamp was sealed off the vacuum system, of course, by immersing the side limb with which the lamp had been provided into a coolant. The white discharge appeared and the molecular background became objectionable at temperatures of the order of −60 °C, which corresponds to a bromine vapor pressure of slightly less than 1 mm of mercury.

Despite this procedure, the molecular background appeared throughout the visible region of the spectrum on plates bearing long exposures. It is thus possible that some of the fainter lines in the appended table of wavelengths, especially in the region from 5000 A to 7000 A, actually are characteristic of molecular, rather than atomic bromine. Further, bromine exhibits continuous emission which begins strongly at about 3800 A and extends to shorter wavelengths although on one set of plates of emulsion type EK I–F exposed for 5 hours this continuous emission already began faintly at about 4400 A. Above that wavelength, discrete molecular lines appear on these plates.

Within this region of continuous emission, atomic emission lines could still be observed and measured. It was discovered, however, that it was more satisfactory in the region below 4000 A to reduce the pressure in the lamp by immersing the side limb in a mixture of solid carbon dioxide and alcohol at about −70 °C. This procedure had the result that the continuous emission spectrum disappeared and was replaced by a weaker background of discrete molecular lines upon which the atomic emission lines, now enhanced, could more easily be identified and measured.

All of the spectrograms bore exposures to an electrodeless lamp containing thorium or iron, or to the iron arc, to serve as standards in the determination of bromine wavelengths. In the first series of observations, the plates were reduced by linear interpolation with a correction curve, whereas in the second series, the micrometer readings of the bromine lines and of the standard lines on each plate were punched on cards so that the computation of bromine wavelengths could be accomplished by an IBM-704, which adjusted all wavelengths by least squares to fit a fourth degree equation.

## 3. Results

In the table are listed the wavelengths of the lines of Br I, together with their character and estimated intensities. It includes all the lines observable by photography in air, since there are no lines of Br I between 1700 A and 3300 A. The given wavelengths of most lines are weighted means of from two to eight different measurements. The character of a line is indicated by inserting the following symbols after the intensity of the line:
blblendcpartially resolved hyperfine structureddouble linehhazy, diffuseHvery hazy, very diffusewwidened line, probably owing to unresolved hfsWvery wide line

The intensities are visual estimates on an approximately linear scale where the faintest line is assigned an intensity 1. It must be emphasized that these relative intensities are valid only over small wavelength ranges, and no attempt has been made to adjust the values in accordance with the varying sensitivity of the photographic emulsions. In the case of lines which are separated by just a few angstrom units and which thus all appeared at once in the field of view of the microscope of the comparator used to measure the spectrograms, finer variations of intensities have been indicated, which, however, are meaningless over a wider range. For example, a group of four lines in the field, all of which appear with an intensity of about 200 when compared with other lines on the same plate, might be assigned intensities 180, 195, 205, and 200, to give an approximate index of their own relative intensities.

## Figures and Tables

**Table 1:** Wavelengths and intensities of Br I

Wavelength	Intensity

*A*	
3325.307	10 *w*
3347.990	4
3348.566	15
3400.050	40 *c*
3402.420	20 *c*
3409.740	20 *c*
3418.888	15 *w*
3425.577	60
3428.605	15
3429.809	8
3472.200	30 *c*
3496.366	5
3497.430	1
3516.144	40
3541.173	100
3556.530	40 *c*
3557.461	250
3558.616	75
3563.706	50
3564.480	15 *c*
3579.081	40
3581.712	15
3589.818	100
3599.962	100
3606.518	20
3608.864	7
3644.190	10 *c*
3646.620	100 *c*
3658.486	2 *h*
3659.970	20
3735.800	500
3745.419	50
3751.287	10
3753.351	5
3756.032	30
3757.265	5
3761.746	2
3761.847	1
3770.880	150 *c*
3794.030	900 *c*
3798.805	5 *w*
3810.950	1 *h*
3811.440	30 *h*
3815.650	1200
3828.505	700
3829.750	200 *c*
3844.035	100
3847.360	2 *h*
3850.619	10
3850.740	1
3851.654	3
3854.702	90
3896.653	200
3899.767	25
3905.850	20 *c*
3909.385	175
3913.560	12
3917.810	200 *c*
3929.196	5
3934.090	125 *c*
3942.026	2 *h*
3943.776	2
3943.835	8
3945.565	4
3969.430	1 *c*
3973.967	5
3991.363	300
3992.363	1500
3993.177	5
3998.492	35
3999.070	200
4000.599	2 *w*
4009.310	2 *c*
4012.570	75 *c*
4016.453	1 *h*
4018.316	2
4021.772	250
4033.762	1 *h*
4037.324	500
4056.420	35 *c*
4057.020	30
4058.406	75
4064.150	4
4075.503	50
4076.956	75
4083.149	50
4094.643	35
4106.352	25
4113.132	4
4114.165	25
4124.233	75
4143.974	600
4157.425	250
4164.158	4
4164.270	15
4168.115	10
4175.786	750
4179.322	100
4191.935	50
4196.468	50
4199.473	35
4202.487	450
4220.701	5 *h*
4220.859	2
4231.643	4
4240.366	40
4242.497	2 *h*
4248.414	50
4250.818	40
4250.924	15
4260.625	40
4261.964	3
4334.54	20
4342.45	2
4358.33	1
4365.14	2000
4369.22	75
4391.60	800
4399.73	450
4404.57	10
4412.49	100
4423.03	200
4425.14	1500
4427.20	40
4431.59	1
4441.74	10000
4446.08	15
4466.21	200
4469.98	150
4471.67	80
4472.61	10000
4475.87	15
4477.72	20000
4479.59	15
4490.42	1000
4513.44	3000
4513.82	150
4525.59	15000
4528.61	400
4529.79	600
4575.74	3000
4592.16	200
4614.58	2500
4640.92	40
4641.02	70
4643.52	900
4693.56	300
4703.42	50
4706.36	12 *h*
4720.03	100
4722.97	9
4727.05	4
4752.28	2500
4757.19	2
4762.04	4
4763.84	5
4765.18	8 *h*
4765.63	250
4775.20	750
4780.31	4000
4785.19	1600
4796.85	5
4802.67	250
4803.47	5
4804.64	4 *h*
4806.03	6
4806.57	7
4807.61	350
4809.46	5
4812.09	6
4812.21	6
4814.80	7
4817.21	3
4818.71	7
4834.46	500
4848.39	25
4860.04	250
4906.49	10
4920.98	300
4921.80	10 *w*
4954.73	300
4979.76	4000
4983.25	75
5002.72	500
5029.37	200
5063.74	40
5083.83	6 *w*
5092.60	4
5099.79	20
5138.55	10
5139.47	10 *w*
5148.78	10
5150.47	6 *h*
5220.71	8
5222.32	12
5226.91	20
5241.43	5
5245.12	350
5261.80	5
5285.22	50
5297.32	15
5310.04	5 *h*
5314.18	10
5315.85	8
5317.25	20
5318.83	7
5323.20	12
5328.92	200
5329.30	12
5337.10	40
5345.42	600
5348.29	10
5354.67	120
5356.74	12 *w*
5364.19	300
5365.20	5 *h*
5370.34	300
5379.13	15
5382.96	120
5384.27	8
5393.58	7
5395.50	1500 *c*
5414.26	4
5420.43	13
5420.80	18
5424.61	8 *h*
5432.47	60
5434.22	7
5450.09	550
5453.03	90
5455.16	60
5455.96	70
5463.72	80
5466.22	1200
5469.76	15
5470.90	5
5504.46	35 *h*
5512.98	5 *h*
5520.86	20
5522.53	90
5529.01	175
5532.22	100
5536.37	300
5544.72	6
5546.90	20
5558.13	40
5558.40	10
5588.17	160
5590.56	50
5597.26	15
5602.41	8 *h*
5604.96	4
5609.83	10 *h*
5611.62	125
5627.24	200
5633.97	140
5637.27	130
5640.86	35
5645.31	30
5645.96	45
5667.75	9
5667.92	8
5685.77	15 *W*
5697.29	18
5705.74	85
5709.44	9
5716.26	225
5721.11	100
5722.97	40 *h*
5764.66	45
5771.30	5
5777.69	10
5779.97	4
5783.32	500
5784.02	30
5793.98	100
5801.50	40
5803.82	30
5805.04	30
5809.59	250
5819.56	80
5821.45	12
5826.07	5
5827.07	10
5828.51	150
5828.89	7
5830.39	400
5833.39	900
5836.84	150
5852.08	1800
5861.20	120
5864.82	90
5867.05	60
5869.47	18
5877.15	50 *w*
5886.87	60 *cw*
5889.87	60
5894.28	18
5898.32	8
5898.51	8 *d*
5898.96	90
5905.45	900
5905.66	80
5909.31	250
5937.85	90
5940.48	1600
5943.71	140
5945.50	600
5950.32	750
5953.92	750
5982.90	12
5983.35	5
5985.32	125
5992.89	100
5999.73	95
6007.96	200
6018.17	200
6022.66	30
6025.24	10 *h*
6048.15	5
6057.87	200
6059.74	5
6061.46	5
6062.88	4
6064.35	200
6067.36	5
6095.74	700
6107.69	125
6111.10	80
6116.19	60
6118.80	300
6122.14	2400
6126.53	30 *W*
6132.71	800
6134.70	75
6137.49	150
6141.04	100 *w*
6142.73	100
6146.00	25 *cw*
6148.60	40000
6151.10	50 *d*
6158.19	750
6177.39	2000
6184.09	200
6199.74	50
6203.08	900
6204.35	90
6204.49	90
6205.40	120
6208.26	100
6216.71	5
6225.51	65
6235.87	10
6244.39	400
6248.24	200
6251.32	300
6253.69	400
6282.46	600
6284.69	8
6290.13	550
6296.71	700
6301.36	275
6315.12	5
6331.99	300
6335.48	1500
6336.48	500 *cw*
6337.85	60
6343.79	35 *w*
6345.30	500
6349.82	500
6350.73	60000
6371.60	200 *w*
6392.57	35 *W*
6394.67	7
6398.03	30
6399.99	10 *W*
6405.65	15 *cw*
6410.32	2500
6418.28	45
6418.97	35 *cw*
6426.30	500
6435.81	15
6436.60	10
6438.02	600
6458.92	25 *h*
6462.32	500
6470.41	200
6475.23	100 *h*
6483.56	1800
6483.96	35
6488.62	800
6493.80	20 *h*
6501.50	4
6514.32	12
6514.62	1000
6531.39	70 *bl* *with* Cl
6532.29	600
6541.30	600
6544.57	20000
6548.09	1500
6551.57	12
6559.80	50000 *cw*
6571.31	1000
6574.29	15
6576.24	10 *W*
6579.14	1800
6579.36	300
6582.17	20000
6584.14	600
6589.62	10 *w*
6604.80	15
6613.05	4
6620.47	1500
6621.44	8
6624.26	20
6631.62	50000 *cw*
6635.10	60
6636.62	150
6639.57	40
6645.17	40
6646.59	120
6653.82	15
6666.93	150
6672.15	600
6676.54	15
6676.72	8
6682.28	20000
6684.22	100 *cw*
6687.33	90
6688.08	90
6690.35	120 *cw*
6692.13	10000
6694.62	10
6700.71	60 *h*
6702.07	110
6706.79	60
6712.12	30
6713.06	125
6714.86	90
6719.97	8
6720.68	75 *h*
6723.65	400
6728.28	8000
6738.61	100
6739.66	8
6740.83	5
6752.67	6
6760.06	2000
6761.92	25
6765.12	8
6771.95	175
6774.63	30 *h*
6778.57	60
6779.48	2000
6785.23	10
6785.74	900
6786.74	2200
6787.34	175
6787.77	8
6790.04	6500
6791.48	1600 *c*
6801.35	60
6816.72	50
6820.39	800 *c*
6826.02	400
6840.62	175
6844.82	5
6845.24	40
6845.30	40
6846.27	150
6858.22	45
6859.43	20
6861.15	1800
6875.22	20 *w*
6887.99	12 *h*
6888.73	80 *h*
6895.65	1 *w*
6904.95	400
6922.86	30
6923.04	1
6927.34	8
6929.78	400
6936.76	20
6962.99	10
6971.96	250
6992.25	5
6992.83	5
6993.31	20
7005.19	10000
7011.53	50
7015.15	75
7024.70	20
7026.53	2
7031.36	15
7054.88	50
7058.38	200
7061.71	1
7066.33	4
7076.71	5
7082.34	2
7082.63	3
7091.12	4
7101.80	5 *h*
7111.62	200 *cw*
7113.22	50
7113.60	100
7117.59	15
7120.78	10
7120.87	10 *c*
7133.95	5
7138.05	2
7138.19	4
7142.25	500 *c*
7145.56	2
7147.06	4
7149.08	15
7150.00	8
7150.30	75
7153.13	5 *h*
7160.74	15
7162.10	750
7172.22	25
7175.74	5 *H*
7177.89	5 *h*
7184.30	300
7194.40	15 *cw*
7214.95	5 *c*
7217.78	20
7222.31	50
7232.45	100
7236.86	7
7247.29	5 *h*
7255.47	10 *cw*
7255.63	15 *W*
7257.40	4
7257.82	10
7260.45	2000
7261.46	20
7262.40	25
7265.16	5
7272.58	3 *w*
7284.41	5
7288.40	150
7291.92	1
7305.03	75
7310.45	4
7311.48	100 *d*
7319.44	50 *cw*
7323.35	10
7329.20	10 *hw*
7333.72	50 *c*
7344.53	100
7348.51	10000
7378.41	20 *d*
7383.69	5
7385.54	4 *h*
7420.69	2
7425.85	750
7452.11	60
7453.41	70
7458.22	50 *h*
7461.84	70
7464.67	8
7495.46	15 *hw*
7497.79	5
7512.96	40000
7532.76	50
7535.78	400
7551.49	500 *cw*
7559.97	70
7569.07	500
7570.85	550
7575.22	15 *w*
7582.55	10 *h*
7582.98	100 *h*
7586.59	25
7591.59	1600
7594.50	25
7595.06	1800
7598.00	4
7604.02	70
7606.26	120
7607.37	400 *cw*
7616.39	2000
7617.90	20 *cw*
7632.85	50
7641.62	600
7647.67	40
7652.82	150 *cw*
7663.51	100 *cw*
7680.71	10 *hw*
7681.74	20
7685.08	50
7698.71	35
7699.56	80
7704.32	60 *h*
7706.68	15 *hw*
7708.38	8
7709.34	15 *h*
7709.54	15 *h*
7711.68	5
7713.28	200
7713.53	45
7715.30	500 *cw*
7721.45	300
7726.16	125 *cw*
7733.61	900
7734.60	25
7742.00	1
7795.00	15
7803.02	30000
7807.66	125 *cw*
7821.09	30
7827.23	1200
7835.09	30
7841.87	30
7843.58	600
7844.04	150 *d*
7869.52	10
7881.52	4000 *c*
7889.85	600
7903.66	50
7905.69	50
7925.81	2500
7929.68	250
7932.97	30 *W*
7938.68	30000 *c*
7940.04	60
7947.94	3000
7950.18	3000
7961.33	350
7966.94	900 *c*
7978.44	8000 *bl*
7978.57	10000 *bl*
7987.12	30
7989.94	30000
7997.01	80
7998.74	175
8004.35	25
8008.87	30 *h*
8009.36	75
8010.00	800 *cw*
8014.72	500 *cw*
8021.70	500 *cw*
8022.51	800
8023.13	20
8023.91	900
8026.45	5000 *c*
8028.21	18
8028.79	400
8029.66	500 *cw*
8030.91	250 *h*
8033.40	40
8034.94	250 *cw*
8037.32	40
8037.63	60
8050.40	15
8066.36	200
8072.87	300
8101.20	150
8111.55	50
8113.02	20 *h*
8131.52	30000
8137.88	150
8142.79	40 *h*
8152.65	1000 *c*
8153.75	10000
8153.98	25000
8166.30	175
8170.19	200 *d*
8172.07	200
8175.70	20
8175.57	25
8179.62	40
8179.98	100
8183.49	450
8189.91	1 *hw*
8190.05	1 *hw*
8190.25	40
8197.71	175 *h*
8210.48	3 *h*
8215.11	200
8237.94	500
8246.86	5000
8242.97	30
8252.38	30
8253.87	35
8258.32	300
8264.96	15000
8272.45	75000 *c*
8280.23	100
8280.75	100 *w*
8291.05	900
8293.56	100
8304.59	10
8308.48	20
8313.09	10
8334.70	20000
8343.69	10000
8357.53	10
8359.05	5
8361.71	300
8362.38	100 *h*
8367.19	15
8369.00	20
8372.77	150
8378.63	100 *w*
8384.02	1200
8387.97	100
8389.73	300
8392.76	75
8397.61	50
8402.07	20
8408.67	250
8410.79	10 *w*
8411.61	5 *w*
8421.88	4
8428.76	75
8430.50	50
8432.49	10
8434.83	150
8435.33	75
8441.25	100
8446.55	40000
8454.25	75
8463.32	4
8470.62	100
8471.51	500
8477.45	4000
8484.40	30
8491.52	4
8503.78	400
8513.38	1500
8515.68	100
8529.38	8 *h*
8557.73	1000
8560.57	300
8565.28	200
8566.28	1000
8578.84	400
8580.00	20
8592.32	250
8595.49	50
8595.66	50
8603.69	100
8604.06	20 *hw*
8606.68	20
8608.97	30 *hw*
8610.48	100
8611.83	250
8612.48	75
8625.39	750 *c*
8628.84	200
8638.66	20000
8655.10	100
8658.84	10
8668.84	300
8698.53	4000
8708.68	75
8708.95	100
8725.33	500
8726.75	100 *c*
8741.63	50
8742.98	75
8749.09	10
8757.22	10
8760.46	700
8764.19	500
8775.70	250
8779.16	50
8793.47	10000 *c*
8801.03	150
8803.40	100
8804.77	20
8807.57	100 *c*
8808.85	500
8810.57	10
8819.96	15000
8825.22	25000
8833.32	175
8839.98	20
8842.84	125
8861.69	85
8869.64	300
8870.45	200
8877.89	90
8878.41	90
8882.86	30
8883.46	15
8886.35	40
8888.98	4000
8890.27	50
8895.21	20
8895.88	150
8897.62	30000
8909.72	300
8932.40	6000
8933.29	25
8936.96	15
8944.84	50
8945.19	15
8949.38	1800
8955.44	30
8964.00	9000
8972.82	350
8979.59	200
8984.56	100
8995.22	50
9005.21	50 *h*
9012.23	20
9029.94	300
9045.08	40
9066.26	400
9078.64	700
9079.29	125
9084.29	25
9086.81	100
9107.39	10
9123.17	50
9129.31	70
9147.05	75 *h*
9151.49	4
9166.06	30000
9173.62	15000
9178.16	20000
9183.14	30
9193.46	20
9197.16	50
9201.22	80 *w*
9201.78	80
9202.48	80
9220.55	200
9221.20	35
9226.57	15
9229.11	5
9254.06	70
9254.39	30
9265.42	40000
9282.78	100
9288.56	200
9314.85	30
9320.86	15000
9325.32	20
9352.55	175
9354.56	15
9369.96	100
9379.22	30
9399.57	5
9400.18	90
9409.56	20
9438.22	80
9460.14	500 *d*
9470.67	20 *h*
9475.08	80
9476.32	30
9477.48	60
9488.66	120
9495.30	50
9498.32	20
9503.78	6 *w*
9504.53	3
9507.03	75
9517.27	100
9543.04	40
9552.65	175
9560.58	6
9570.32	2
9588.60	300
9590.33	20
9598.78	60
9603.62	35
9604.30	6
9621.02	35 *h*
9623.33	15
9625.17	8
9627.54	4
9631.29	60
9635.05	20
9662.00	40
9662.99	20
9710.64	12
9717.44	40
9719.18	600
9725.39	40
9731.73	350
9732.86	50
9740.38	15
9745.83	100
9751.41	90
9793.43	6000
9810.63	60
9817.98	60 *bl*
9818.15	70 *bl*
9828.34	15
9844.13	7
9864.92	2
9896.37	10000
9944.04	400 *c*
9945.19	1
9962.04	35 *c*
9963.86	1
10001.92	10
10002.15	5 *h*
10025.20	*4 h*
10045.67	3 *h*
10056.86	15
10061.57	25 *d*
10079.72	10
10085.97	35
10087.92	1
10088.27	10
10102.79	12
10107.76	150 *d*
10113.72	2
10119.17	3
10128.76	4
10139.04	3000
10174.72	12
10175.44	20
10183.98	300
10184.45	200
10197.58	18
10208.88	300
10225.20	1
10231.58	8
10232.36	15
10237.11	75
10237.72	6000
10243.28	3
10248.24	3
10265.85	15 *h*
10293.63	50
10299.62	1000
10305.96	10
10310.50	800
10310.72	600
10310.91	700
10312.87	40
10314.01	8
10320.06	15
10324.93	3
10329.99	100 *cw*
10374.35	12 *cw*
10377.61	1500
10390.71	175
10392.49	25
10415.05	12 *w*
10457.90	30000
10483.31	8
10505.02	8
10507.87	12
10513.64	1
10529.48	3 *h*
10539.14	6
10554.72	1
10566.12	250
10566.85	100
10569.62	2
10591.48	20 *w*
10608.64	40
10616.27	1
10619.62	1
10624.28	1
10629.43	18
10634.04	1
10638.80	15
10660.76	3
10718.80	100
10723.07	15
10742.09	1000
10742.43	100
10747.17	20
10753.88	200 *cw*
10755.86	3000
10757.76	6
10795.01	10 *cw*
10798.06	25
10804.38	3
10810.04	300
10815.63	6
10840.04	500 *d*
10869.65	100
10871.58	90
10891.37	250
10892.74	100
10896.75	200
10908.58	60 *w*
10973.40	500
10978.52	40
10978.93	300
10982.25	100
10991.07	2
10997.78	600
10998.23	400
11007.36	10
11010.38	600
11012.27	5
11013.17	20
11024.19	10
11039.75	60
11045.69	800 *d*
11047.15	10
11047.57	17
11074.04	40
11093.46	250
11094.19	100
11096.54	20
11100.59	1
11101.30	1
11161.68	1 *w*
11194.94	100
11197.00	2
11197.11	3
11225.04	250
11247.46	2
11286.92	2
11292.69	2
11297.98	2
11316.92	10 *w*
11325.97	1
11330.17	3
11350.02	65
11367.21	4 *w*
11367.88	4 *w*
11436.18	3
11437.59	9
11447.25	18
11464.56	35
11501.27	1
11508.75	30
11583.66	10
11597.58	1
11631.30	2
11636.05	1
11640.91	1
11666.17	20 *d*
11742.82	400
11810.27	1
11819.31	2
11833.03	1
11870.75	4
11883.89	4
11902.60	5
11903.57	8
11928.09	8
11990.51	15
12088.15	2
12285.46	2
12303.82	35
12349.26	1
12354.35	1
12368.85	1 *w*
12450.44	1
12809.50	1
12826.01	1
12965.11	4

## References

[1] Kiess CC, Corliss CH (1959). J Research NBS.

[2] Martin WC, Corliss CH (1960). J Research NBS.

[3] Kiess CC, deBruin TL (1930). BS J Research.

[4] Corliss CH, Bozman WR, Westfall FO (1953). J Opt Soc Am.

